# Spatial variation and metabolic diversity of microbial communities in the surface sediments of the Mariana Trench

**DOI:** 10.3389/fmicb.2022.1051999

**Published:** 2022-12-05

**Authors:** Fangzhou Wang, Yue Zhang, Hongmei Jing, Hao Liu

**Affiliations:** ^1^CAS Key Lab for Experimental Study Under Deep-Sea Extreme Conditions, Institute of Deep-Sea Science and Engineering, Chinese Academy of Sciences, Sanya, China; ^2^University of Chinese Academy of Sciences, Beijing, China; ^3^HKUST-CAS Sanya Joint Laboratory of Marine Science Research, Chinese Academy of Sciences, Sanya, China; ^4^Southern Marine Science and Engineering Guangdong Laboratory (Zhuhai), Zhuhai, China

**Keywords:** microbial community, carbon source utilization, diversity, potential metabolic functions, Mariana Trench

## Abstract

Mariana Trench represents the deepest and one of least explored biosphere on Earth, and its carbon sources include euphotic sinking, lateral transportation and diffusion from underlying crust, etc. By far the spatial variation of microbial community with associated organic carbon degradation potential in the surface sediments of the Mariana Trench were still largely unknown. Based on the high-throughput 16S rRNA amplicon sequencing, significantly different microbial community structure was overserved between the shallow (<10,000 m) and deep stations (>10,000 m), which could be explained by spatial variation of Chloroflexi, Proteobacteria and Crenarchaeota, with sampling depth and total organic carbon (TOC) content as the environmental driving forces. During the 109-day incubation with Biolog EcoPlate™ microplate, polymers and carbohydrates were preferentially used, followed by amino acids and carboxylic acids, and microbial metabolic diversity was significantly different between the shallow and deep stations. The metabolic diversity of microorganisms at most shallow stations was significantly lower than that at deep stations. This could potentially be attributed the metabolic capabilities of different microbial groups with varied ecological niches, and reflected the initial preference of carbon source by the nature microbes as well. Our study obtained a rough assessment of physiological and taxonomic characteristics of the trench sediment microbial community with polyphasic approaches. Distinct microbial structure and potential carbon metabolic functions in different sampling depths might led to the differentiation of ecological niches, which enable various microorganisms to make full use of the limited resources in the deep sea, and provided a research basis for further exploration of the carbon cycle in different deep-sea regions.

## Introduction

Deep-sea ecosystems represent the largest biomes and play fundamental roles in global biogeochemical cycles (Worden et al., 2015). Deep-sea microorganisms are highly adaptable to this specific environment and have developed unique diversity and metabolic mechanisms (Wang et al., 2013). Although chemoautotrophic microorganisms are the primary producers of deep-sea habitats, heterotrophic microorganisms are the vital consumers of organic matters. Particulate organic matters (POM) settled from the euphotic zone were composed of phytoplankton debris, zooplankton and animal fecal balls, aggregates, marine snow, transparent polymeric particles and colloidal particles (Azam and Malfatti, 2007), and were proposed as a major carbon source for seafloor microorganisms (Xu et al., 2018). In addition, the supply of organic matter from the above water column and lateral transportation from margins would affect the diversity and metabolic function of microbial community as well (Bochdansky et al., 2010). Utilization of different carbon sources by several marine bacterial isolates has also been reported previously, for example, three strains of *Colwellia psychrerythraea* could grow on 25 of the 190 carbon sources tested (Techtmann et al., 2016). Generally, microbial communities and their utilization on the available carbon source in the deep-sea ecosystems deserve further investigation.

Different microbial groups have different preference on carbon sources, which in turn affects their metabolic activities and community composition. Therefore, it is essential to study the link between the community diversity and their metabolic diversity for the natural microbial communities. Recently, Biolog EcoPlate™ (Biolog Inc., CA, United States) was used to evaluate community level physiological profiling from a variety of environmental matrices, including Canadian Arctic (Sala et al., 2008), Antarctica (Kenarova et al., 2013), Mediterranean (Stabili et al., 2017), and the western coast of the Indian Arabian (Kumar et al., 2019). This method could better identify microbial carbon source utilization, and combined with high-throughput sequencing technology would be useful for linking community structure with the actual metabolic function (Kiersztyn et al., 2019). However, this method has been seldomly applied to the deep-sea microbes (Sinha et al., 2019), especially in the deepest biosphere such as trenches.

Mariana Trench is the deepest place on Earth, and characterized by high water pressure, complete darkness, low temperatures and oxygen content (Xu et al., 2018). Owing to the funnel structure of trenches, sediments accumulate particularly along the trench axis and vary in terms of quality and quantity with depths (Nunoura et al., 2018). Increased organic matter deposition in the benthic sediments of the Mariana Trench has resulted in enhanced microbial activity and high microbial carbon conversion rates (Glud et al., 2013). The *Flavobacteriia* with carbohydrate catabolism ability (Liu et al., 2019) and heterotrophic bacteria (Zhao et al., 2020) with capability of degrading macromolecules have been isolated from Mariana Trench. In addition, genes associated with degradation pathways of carbohydrate, hydrocarbon and aromatic compounds were revealed from metagenomic studies on the sediments of the Mariana Trench (Chen et al., 2021; Jing et al., 2022). It was clear that by far most studies on microbial carbon metabolism in the Mariana Trench were either based on metagenomic analysis or limited to the physiology of several bacterial isolates, and lack of direct evidence linking the community diversity, structure with microbial carbon source utilization capacity.

In this study, diversity and composition of microbial community with associated carbon metabolism in the surface sediments of the Mariana Trench were investigated by using high-throughput sequencing and Biolog EcoPlate™. Our study emphasized on the significance of polyphasic approach in comprehensive assessment of physiological and taxonomic characteristics of the deep-sea sediment microbial community, and aimed to establish a close linkage between microbial community and their metabolic functional activities.

## Materials and methods

### Sample collection and chemical analysis

**Figure 1:**
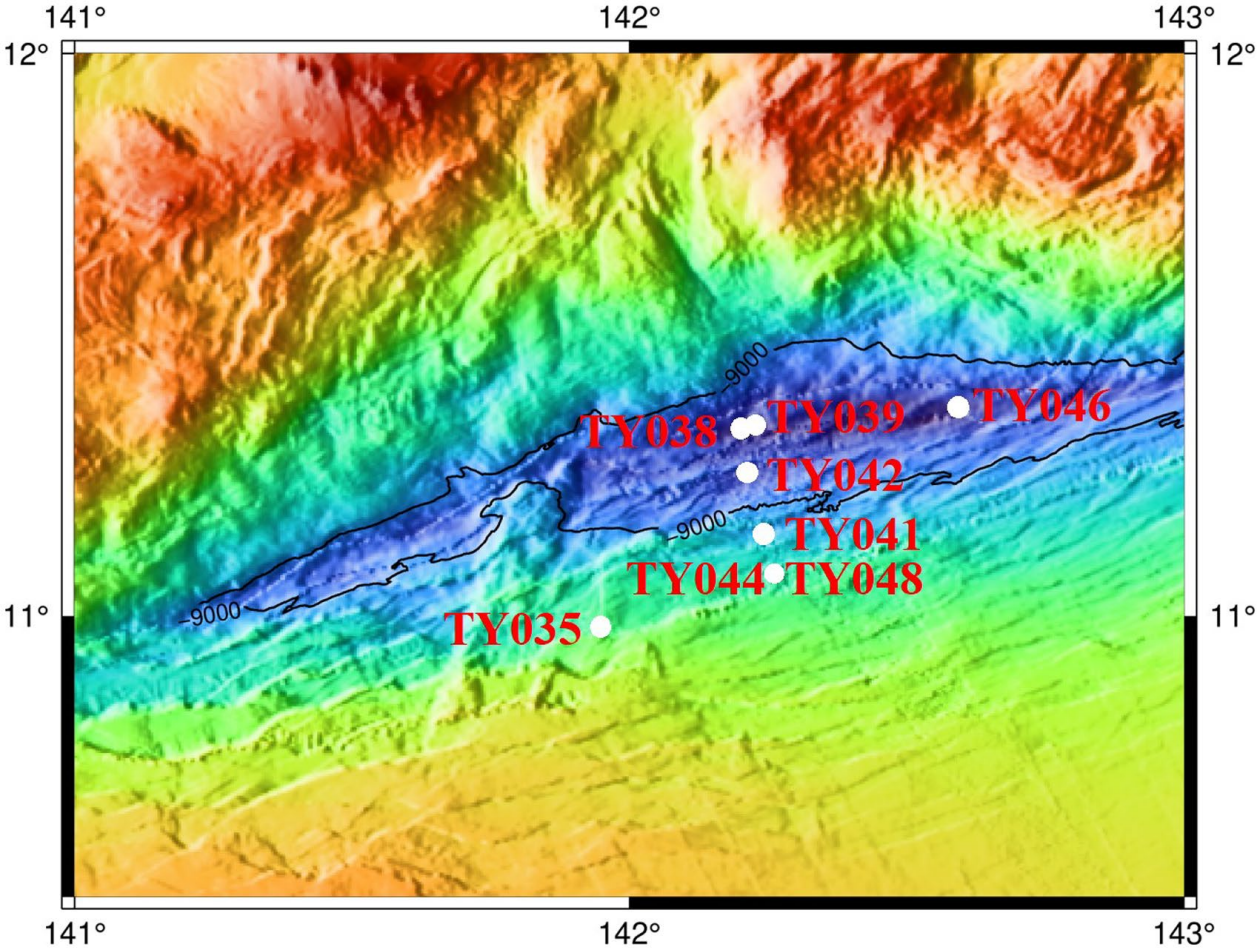
Location of the sampling stations in the Mariana Trench.

Sediment samples were collected using pushcore from eight stations of the Mariana Trench in the Western Pacific Ocean during cruise TS09 from September to October in 2018 by R/V “Tan Suo Yi Hao” (Figure 1). The surface sediment samples (0–4 cm) for subsequent cultivation were stored at 4°C in the dark, and for DNA extraction and measurement of nutrient parameters were immediately stored at −80°C.

Approximate 5 g sediment was used for measurement of chemical parameters at the Institute of Mountain Hazards and Environment, Chinese Academy of Sciences (Chengdu, Sichuan, China). The sediment treated with 1 M HCl was analyzed by a colorimetric auto-analyzer (SEAL Analytical AutoAnalyzer 3, Germany) to detect NO_3_^−^ and NH_4_^+^ concentrations. The content of total carbon (TC) and total nitrogen (TN) were determined using an element analyzer (Elementar vario Macro cube, Germany) after over-drying the sediment at 105°C (Wang et al., 2016). After treated with nitric-perchloric acid, the content of total phosphate (TP) was measured by using the molybdate colorimetric method with a UV2450 (Shimadzu, Japan; Murphy and Riley, 1962).

### Microbial metabolic activity analyses

Biolog EcoPlate™ microplates (Biolog Inc., CA, United States) was used to evaluate community-level metabolic capabilities (Sinha et al., 2019). The EcoPlate contains 31 carbon substrates and a control well without carbon substrate added. It can be grouped into six categories: carbohydrates, polymers, carboxylic acids and amino acids, amines and amides, and each substrate has three replicate wells. The surface sediment store at 4°C (10 g) were placed in 20 ml of 50 mM phosphate buffer (pH 7.0), mixed at 170 r/min for 30 min; centrifuged at 1,500 rpm for 5 min. The supernatant was centrifuged at 10,000 rpm for 20 min. After removing the supernatant, the precipitate was washed twice with sterile phosphate buffer to remove soluble carbon, and suspended in 20 ml of the same solution. Each supernatant containing suspended microorganisms was adjusted to an optical density of 0.25–0.35 at 420 nm with phosphate buffer (Guckert et al., 1996). Aliquots of 150 μl were added per well and microplates were incubated at 4°C in the dark. The absorbance of each well was measured at 590 nm and 750 nm every 24 h for a continuous cultivation of totally 109 days using a Varioskan™ LUX scanning multimode reader (Thermo Scientific, MA, United States). The difference of absorbance value was used to characterize the color change of the microplates by eliminating the turbidity change caused by fungi at 750 nm. While processing the primary data, the average well color development (AWCD) was calculated for each category to compensate for the influence of inoculum density. The AWCD is calculated to determine the utilization of carbon sources and metabolism characteristics. The richness index refers to the number of discoloration wells in each microplate to reflect the utilization of carbon source by each well, and the Shannon index reflects the diversity of the microbial community.


(1) $$AWCD=\frac{\sum R-C}{n}$$

(2) $$H^{\prime }=-\sum p_{{}_{i}}\ln p_{{}_{i}}$$

In Equation 1, R is the absorbance of each well, C is the absorbance of the control well and *n* is the number of substrates present in the particular category (Sinha et al., 2019). In Equation 2, *H*′ is the Shannon index of each well, *p*_i_ is the ratio of the relative absorbance value of the well *i* to the sum of the relative absorbance of the entire plate (Tian and Wang, 2011).

### DNA extraction, PCR amplification and sequencing

DNA was extracted from the surface sediments with the PowerSoil DNA Isolation Kit (MO BIO Laboratories, Inc., Carlsbad, United States), according to the manufacturer’s protocol. The concentration of DNA was determined by Qbuit^®^ 2.0 (life Technologies, United States), and the quality was checked *via* gel electrophoresis. For each station, genomic DNAs were extracted from triplicate samples, and combined for subsequent PCR amplification and sequencing. The V3-V4 region of the 16S rRNA gene was amplified with universal prokaryotic primers of Uni341F (5′-CCTACGGGNBGCASCAG-3′) and Uni805R (5′-GACTACNVGGGTATCTAATCC-3′; Takahashi et al., 2014). For each sample, a unique pair of barcoded was used. PCR amplification was carried out in triplicate using the BIO-RAD C1000 Touch™ Thermal Cycler PCR System in a 20 μl PCR reaction mix, containing 2.0 μl 10 × PCR-MgCl_2_ buffer, 0.5 μl 2.5 mM dNTPs, 0.5 μl MgCl_2_, 0.5 μl forward primer, 0.5 μl reverse primer, 0.2 μl Platinum^®^ TaqDNA ploymerase, 2.2 μl template DNA and 13.8 μl dd H_2_O. Thermal cycling was performed at 95°C for 3 min, followed by 40 cycles at 95°C for 0.5 min, 53°C for 45 s, 72°Cfor 30 s, and a final extension at 72°C for 8 min. A negative control of double-distilled water was also performed during amplification in order to avoid reagent contamination. The paired-end sequencing of the amplicons was then performed with an Illumina HiSeq PE250 sequencer (Novogene Co., Ltd., www.novogene.com).

### Bioinformatics analysis

After sequencing, barcoded and low-quality sequences were removed using QIIME2 (Caporaso et al., 2010). Chimeras detected with UCHIME against the SILVA database release 138 (www.arb-silva.de; Pruesse et al., 2007), and reads presented as a single copy were removed manually. The remaining reads were then clustered into Amplicon Sequences Variant (ASV) by DADA2 (Divisive Amplicon Denoising Algorithm) algorithm. Taxonomy assignment of ASVs that were not affiliated with prokaryotes, as determined from the SILVA database release 138, were further removed (Caporaso et al., 2010). A filtered ASV table of each sample was generated with QIIME2. The richness estimator (Chao1), diversity (Shannon and Simpson), and Good’s coverage were then calculated. For the prediction of functional and metabolic profiles of the prokaryotic community based on the 16S rRNA gene, the open-source R package Tax4Fun (Asshauer et al., 2015) was used with the short reads mode disabled along with the SILVA database 138 as required. Tax4Fun analysis was performed on ASVs of all samples using R (Version 4.1.2). The functional annotation and prediction of metabolic or other putative ecological functions were estimated based on the Tax4Fun-KEGG, then the obtained data was used to make the heatmap of potential metabolic functions.

### Statistical analysis

The non-metric multidimensional scaling (nMDS), based on the Bray–Curtis similarity index, were calculated with PRIMER 5 (Plymouth Marine Laboratory, West Hoe, Plymouth, United Kingdom; Clarke and Warwick, 2001) to show the distribution pattern of prokaryotic communities. An analysis of similarities (ANOSIM), based on the relative abundance of ASVs, was conducted with Paleontological Statistics (PAST) version 3 (Hammer et al., 2001) to test whether there was a significant difference in the microbial community among the sampling sites. To detect potential biomarkers, linear discriminant analysis (LDA) effect size (LEfSe) statistical analysis was performed on the Galaxy platform.^1^ A redundancy analysis (RDA) was performed to identify a possible differentiation of the communities under the constraint of environmental factors, and assess correlations between environmental variables and community variability. The phylogenetic group data were Hellinger transformed, environmental variables were logarithm transformed, and the effects of collinearity (VIF > 10) were removed. The Pearson correlation coefficient was calculated according to the relative abundance of ASVs, to reflect the relationship between dominant phyla in the community.

### Availability of data

All of the 16S rRNA gene sequences obtained from this study have been deposited in the National Center for Biotechnology Information (NCBI) Sequence Read Archive (SRA) under the accession number PRJNA854746.

## Results

### Geochemical characterization of the sediments

Sediment core samples were collected from eight stations across the abyssal-hadal zone (6,957–10,918 m) along the slopes of the Challenger Deep of the Mariana Trench (Figure 1). Among them, Stns. TY035, TY041, TY044, and TY048 were shallower than 10,000 m, Stns. TY038, TY039, TY042, and TY046 were deeper than 10,000 m, and they were grouped as shallow and deep stations, respectively. Stns. TY044 and TY048 were very close to each other. Stns. TY035 and TY046 were, respectively, the shallowest and deepest station and their locations were quite far away from other stations. The nutrient content of each sample was analyzed and calculated based on the weight of the sediment. Pearson correlation coefficient analysis showed that TOC was significantly positively correlated with depth (*p* < 0.05). In general, the geochemical parameters (depth, TC, NO_3_^−^, NH_4_^+^, Moisture) of the deep stations were significantly higher from those of the shallow stations (Supplementary Table S1, ANOSIM, *p* < 0.05).

### Diversity and community composition

In total, 699,657 sequences and 93,177 ASVs were generated from quality reads (Table 1). The maximum number of ASVs was found at Stn. TY042. The highest and lowest diversity (Shannon, Simpson index) and richness (Chao1 index) occurred at Stns. TY039 and TY042, respectively. Generally, the diversity at deep stations was slightly higher than that at other stations.

**Table 1:** The sequencing information and diversity index of sediments collected from the Mariana Trench.

	Station	Latitude (°E)	Longitude (°N)	Depth (m)	Original reads	Quality reads	ASVs	Simpson	Shannon	Chao1	Goods coverage
Shallow	TY035	141.949643	10.979310	6,957	89,124	83,455	9,600	0.90	2.64	24	0.99
	TY044	142.262400	11.074234	7,329	84,340	83,851	10,242	0.91	2.64	22	0.99
	TY048	142.262405	11.074232	7,344	80,758	79,217	9,227	0.90	2.58	20	0.99
	TY041	142.241807	11.143987	8,563	84,748	83,309	10,693	0.89	2.62	23	0.99
Deep	TY042	142.212922	11.254702	10,109	95,720	91,649	15,740	0.85	2.22	18	0.99
	TY038	142.203143	11.331067	10,893	90,919	85,290	12,103	0.92	2.78	27	0.99
	TY039	142.227110	11.340020	10,910	89,254	84,508	13,238	0.92	2.79	24	0.99
	TY046	142.592960	11.371130	10,918	84,794	83,050	12,334	0.91	2.70	22	0.99

**Figure 2:**
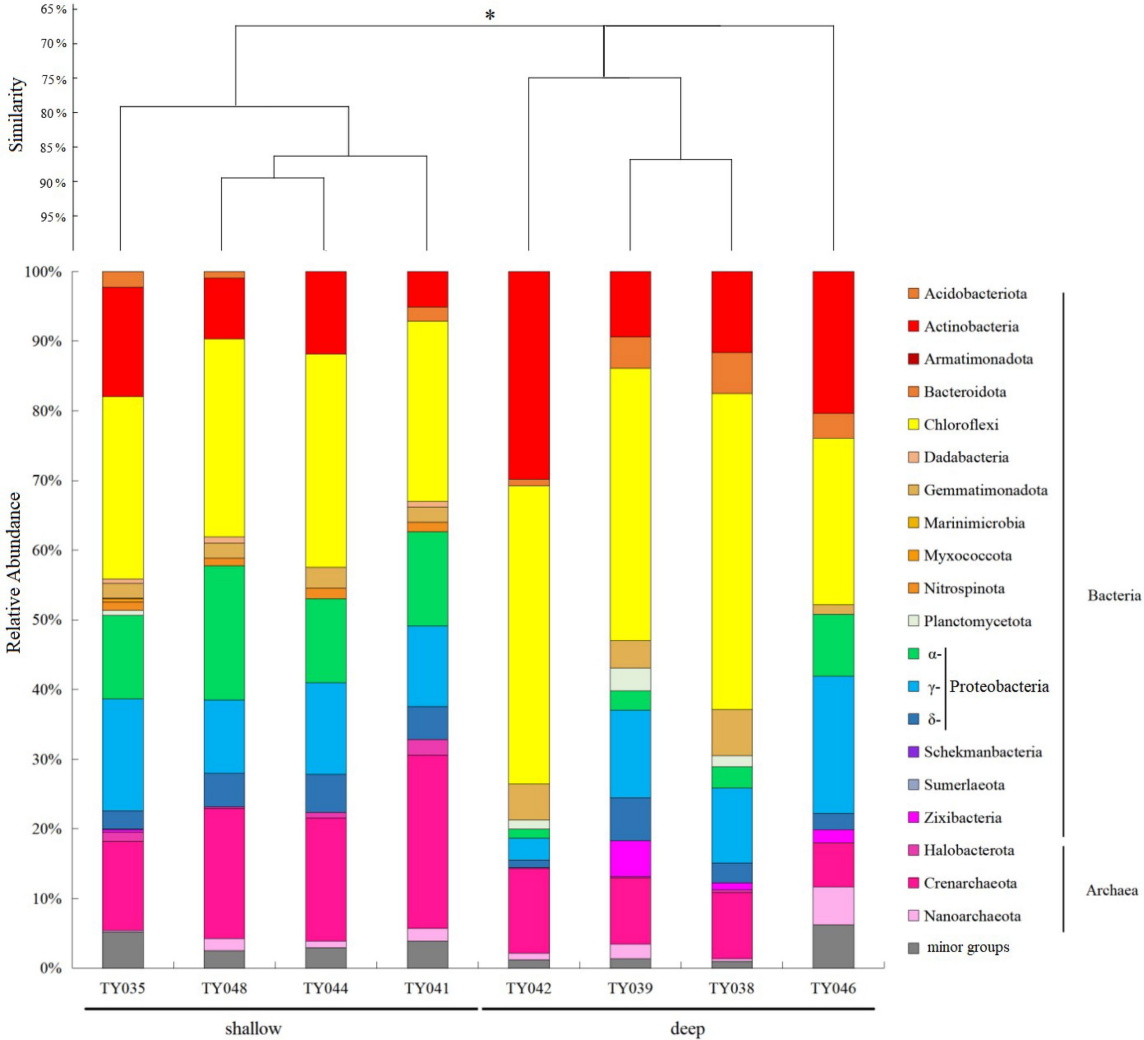
Microbial community structure of different sediment samples with clustering at the phyla level. *p < 0.05. Microbial groups with relative abundance <1% were classified as minor groups.

For the bacterial community, Chloroflexi and Proteobacteria were the two dominant phyla (Figure 2; Supplementary Figure S1). Chloroflexi was mainly composed of SAR202 clade and S085 order, and occupied higher proportions (30%–42%) at deep stations. The highest and lowest relative abundance of Chloroflexi appeared at Stns. TY038 and TY041, respectively. Proteobacteria accounted for higher proportions (23%–34%) at shallow stations, and consist of α-, γ-, and δ-Proteobacteria. Rhodospirillales and Kiloniellales were the dominant α-Proteobacteria groups, whereas Steroidobacterales and SAR324 clade (Marine group B) were the main groups in respective of γ- and δ-Proteobacteria.

As for archaeal community, three phyla, i.e., Crenarchaeota, Halobacterota and Nanoarchaeota, were revealed, with Crenarchaeota as the dominate phylum (Figure 2). Nitrosopumilales as the dominant class of Crenarchaeota had its highest and lowest distribution at Stns. TY041 and TY046, respectively. The relative abundance of archaea at deep stations was lower than that at shallow stations.

### Community similarity, environmental factors, and microbial interactions

**Figure 3:**
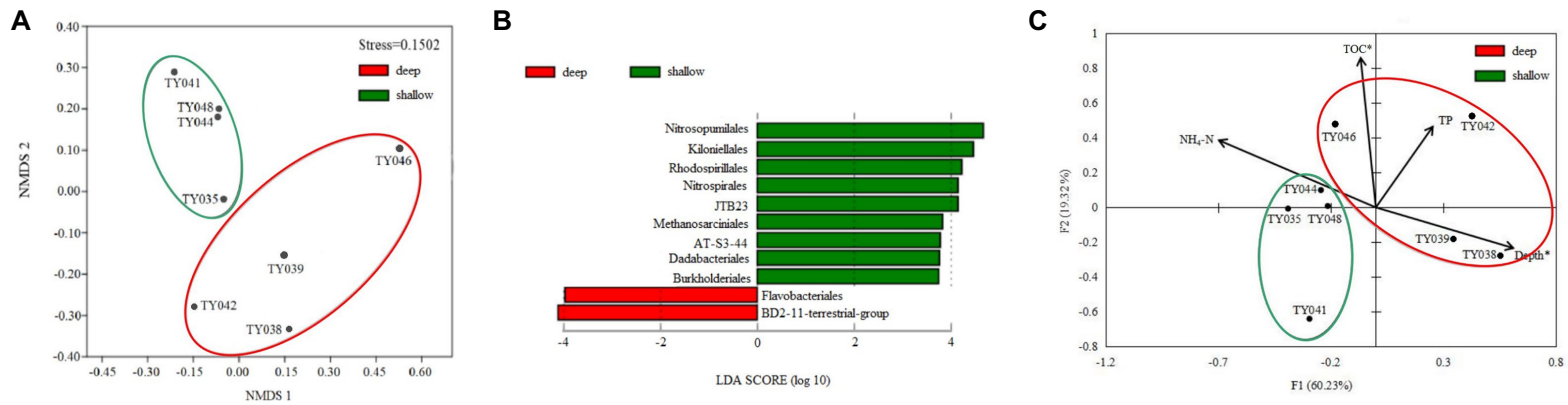
Non-metric multidimensional scaling (nMDS) analysis of microbial communities based on Bray-Curtis distances **(A)**. LEfSe statistical analysis showing linear discriminant analysis (LDA) score between shallow (<10,000 m) and deep (>10,000 m) stations **(B)**. Redundancy analysis integrating the environmental parameters and the relative abundance of prokaryotes taxa at the order level in the different sediment samples **(C)**. (*p < 0.05).

Clustering based on the community structure of prokaryotes demonstrated that those at deep stations were significantly different from that at shallow stations (ANOSIM, *p* < 0.05, Figure 2). nMDS analysis based on the relative of ASVs further proved the significant difference between the two clusters (ANOSIM, *p* < 0.05, Figure 3A). LEfSe statistical analysis showed that the predictive biomarkers at the deep and shallow stations were different. The main predicted biomarkers enriched at deep stations were Flavobacteriales and BD2-11 terrestrial group, while Nitrosopumilales, Kiloniellales and Rhodospirillales were biomarkers at shallow stations (Figure 3B).

RDA analysis based on the microbial communities at the class level demonstrated that the depth and TC content were the key environmental parameters that significantly influenced the microbial community structure (Figure 3C). The first two axes together explained 79.55% of the total variance. Samples from the shallow and deep stations formed two distinct clusters (ANOSIM, *p* < 0.05).

**Figure 4:**
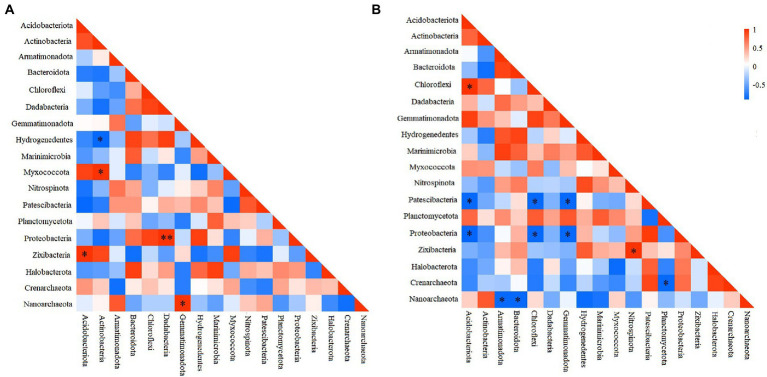
Heatmap of the Pearson correlation for different microbial groups at shallow (<10,000 m) **(A)** and deep (>10,000 m) stations **(B)**. *p < 0.05, **p < 0.01.

To elucidate the interactions among different prokaryotic groups, pearson correlation coefficient analysis was conducted at the phylum level. Correlation heatmap showed that at the deep station, Chloroflexi was significantly positively correlated with Acidobacteriota, but significantly negatively correlated with Patescibacteria, and Proteobacteria (Figure 4A), while Proteobacteria was significantly negatively correlated with Gemmatimonadota and Acidobacteriota (ANOSIM, *p* < 0.05). At shallow stations, significantly negative and positive correlations were found between Hydrogenedentes and Actinobacteria (ANOSIM, *p* < 0.05), and between Proteobacteria and Dadabacteria (ANOSIM, *p* < 0.01), respectively (Figure 4B). Comparatively, significantly negative correlations were more frequently observed between microbial groups at the deep stations.

### Potential metabolic function of prokaryotes

**Figure 5:**
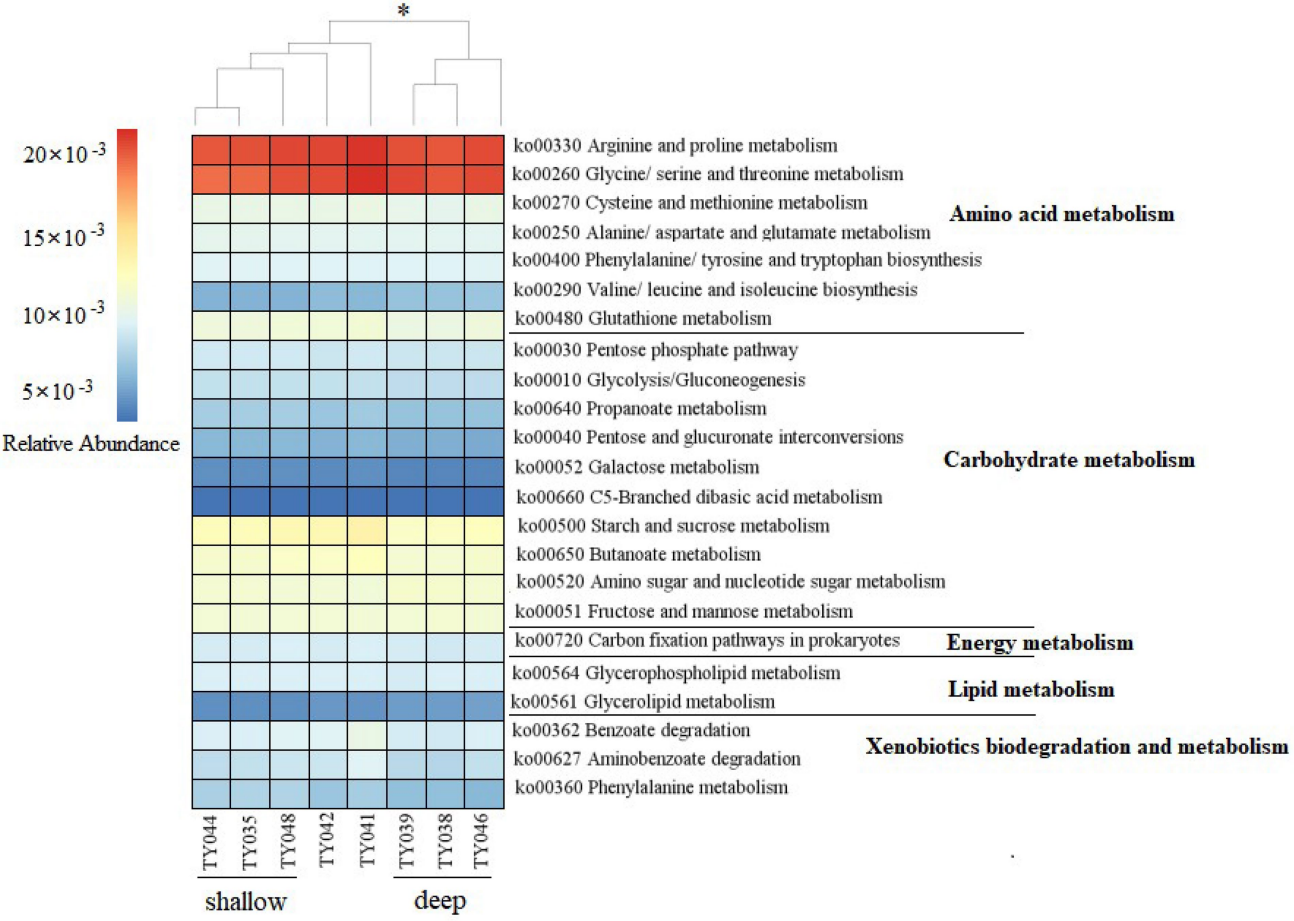
Heatmap of potential metabolism categories of the ASVs base on KEGG metabolism modules. The scale bar represents the relative abundance (×10^−3^) of metabolic pathways, *p < 0.05, ANOISM.

Potential functions of prokaryotes were predicted by Tax4Fun, and functions related amino acids metabolism, carbohydrate metabolism, energy metabolism, lipids metabolism, and xenobiotics biodegradation and metabolism, were prevalent in all samples (Figure 5). The metabolic functions of prokaryotes at deep stations were significantly different from those at the shallow stations (except for Stn. TY042; ANOSIM, *p* < 0.05). Arginine/proline metabolism, and glycine/serine/threonine metabolism were the major types of amino acids metabolism found at all stations. Starch/sucrose metabolism, butanoate metabolism, amino sugar/nucleotide sugar metabolism, and fructose/mannose metabolism were the major types of carbohydrate metabolism. Carbon fixation pathways as an energy metabolism were reveled at all the stations as well.

### Microbial metabolic activity

**Figure 6:**
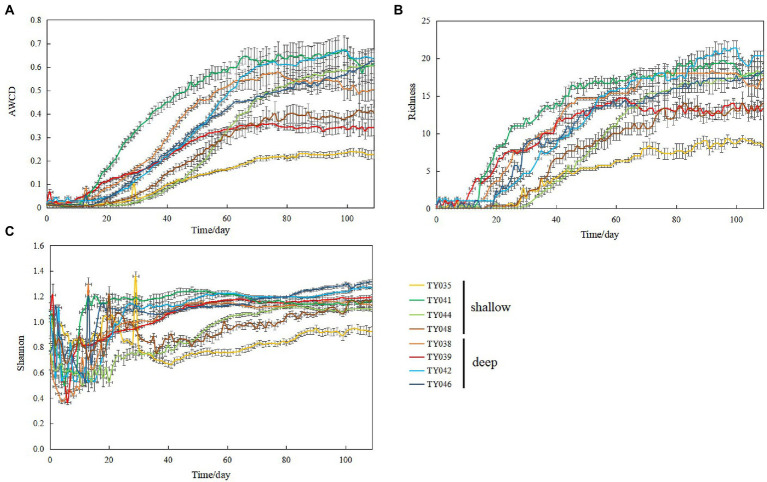
Variations of AWCD **(A)**; richness **(B)**; Shannon **(C)** index for microbial communities in the different sediment samples during the 109-day cultivation in the Biolog EcoPlate™ microplates.

**Figure 7:**
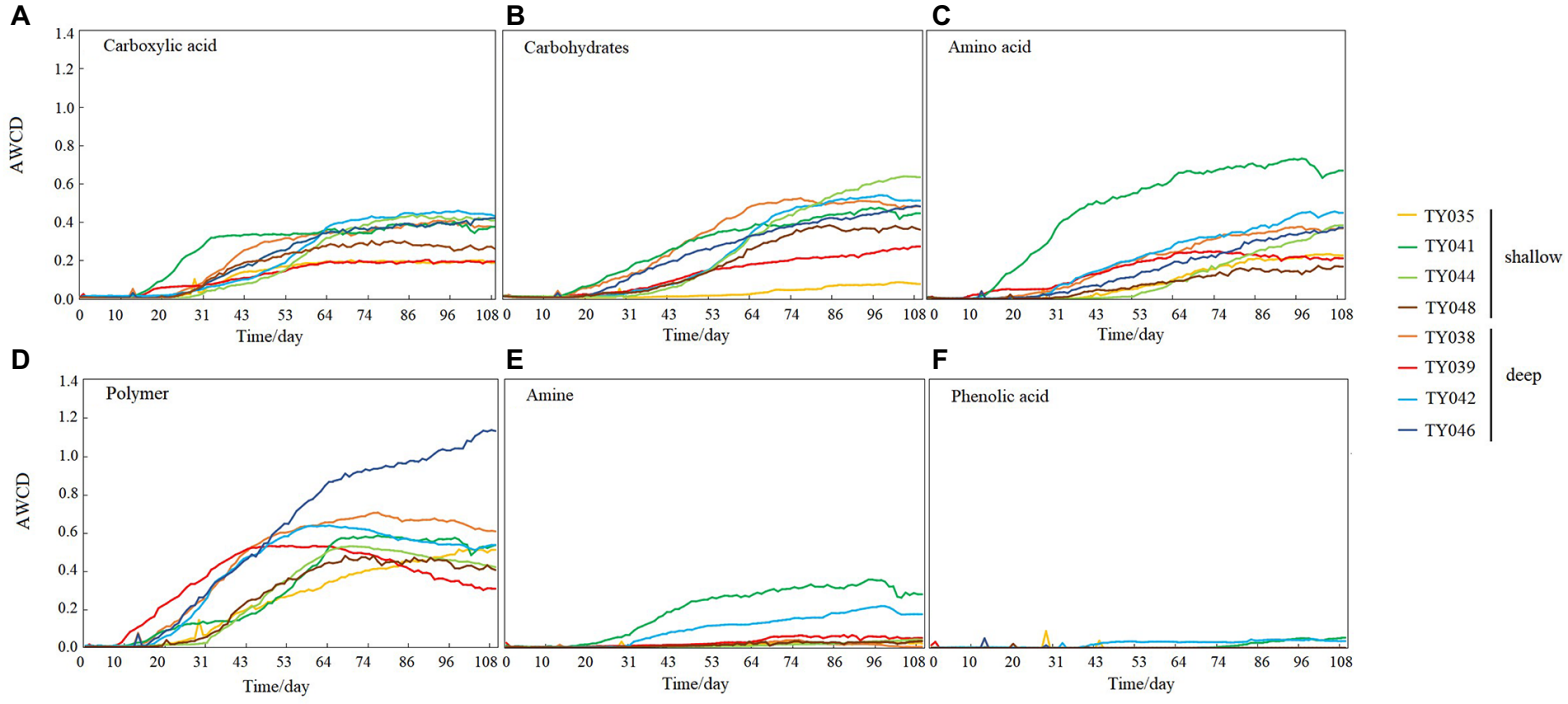
Utilization capability of the six major carbon groups by microbes in the different sediment samples. **(A)** Carboxylic acid; **(B)** Carbohydrates; **(C)** Amino acid; **(D)** Polymer; **(E)** Amine; **(F)** Phenolic acids.

During the 109-day incubation, similar trends of AWCD (Figure 6A) and richness (Figure 6B) index were observed, and both of which entered a stabilization period at about 70 days after a period of exponential increase. As for Shannon index, it varied greatly at first 30 days, and then tended to be stable (Figure 6C). At stabilization period, metabolic activities, represented by richness and Shannon indices at Stns. TY035, TY044, and TY048 were significantly lower than at other stations (ANOSIM, *p* < 0.05), and the lowest values at the end of incubation were found at Stns. TY035. Among the 31 different carbon sources contained in the Biolog Ecoplate™ microplates, polymers were the mostly used carbon category, followed by carbohydrates, amino acids and carboxylic acids, with very little use of amines and almost no use of phenolic acids. Comparatively, microbes at Stn. TY041 had the highest utilization activity to carboxylic acids during the first 60-day incubation, and was later exceeded by those at Stn. TY042 (Figure 7A). For carbohydrates, microbes at Stn. TY041 had the highest utilization activity during the first 45-day incubation, followed by Stn. TY038 in 45–85 days, and Stn. TY044 during 85–109 days (Figure 7B). And for amino acids, microbes at Stn. TY041 had the highest utilization activity during the whole incubation period (Figure 7C). Microbes at Stn. TY039 had the highest utilization of polymers during the first 43-day incubation, and was later exceeded by those at Stn. TY046 (Figure 7D). Except for Stns. TY041 and TY042, amines (Figure 7E) and phenolic acids (Figure 7F) were barely used.

## Discussion

### Geographical distribution and environmental effects

The dominance of Chloroflexi, Proteobacteria and Crenarchaeota in the sediment samples was generally consistent with previous studies conducted in the Mariana Trench (Cui et al., 2019; Chen et al., 2021). Increased proportion of Chloroflexi was found at deep stations, and this might be related to the higher ammonia concentration and organic matter with increasing sampling depth (Nunoura et al., 2018). The overall relative abundance of archaea decreased with sampling depth, was in agreement with previous studies (Nunoura et al., 2018; Chen et al., 2021). Nitrosopumilales, the dominant order in archaea (Supplementary Figure S1), was an ammonium-oxidizing archaea with higher relative abundance at shallow stations (Zhang et al., 2016), generally negatively correlated with *in situ* carbon content (Luo et al., 2015).

In our study, the most important factors that significantly influenced the spatial variation of the microbial community were the sampling depth and TOC content. Sampling depth has been reported as a significant driver of community composition in the Mariana Trenches (Peoples et al., 2018, 2019). Organic matter has been suggested to be an important factor regulating the distribution and abundance of microbial community in marine systems (Hu et al., 2021). The typical V-shaped section of the Marian Trench could act as a funnel to enhance the deposition of organic matter from the steep slope to the bottom (Romankevich et al., 2009), subsequently, the difference of TOC content between shallow and deep stations influenced the microbial community composition.

### Microbial interaction and potential metabolic function

Microbial interactions could lead to a series of competitive or collaborative relationships, and have been proposed as biotic drivers that impact microbial community composition (Calcagno et al., 2017; Hunt and Ward, 2020). At shallow stations, more positive relationships were detected, suggesting that there might exist cross-feeding, co-colonization and niche overlap, whereas widespread existence of negative correlations between microbes at deep stations suggest a possible competition relationship among microbial groups (Faust and Raes, 2012) in the Marian Trench. This depth heterogeneity might be due to different accessing strategies of organic matter by heterogeneous microorganisms (Teske et al., 2011). And the negative correlation of microbial groups has been frequently observed as ecological strategy in the extreme environments such as Arctic deep-sea and hydrothermal vent (Bienhold et al., 2012; Flores et al., 2012).

Carbohydrate and amino acid metabolism as the two major potential metabolisms predicted by Tax4Fun, consisting with the results of Gulf of México in 3,500 m (Moreno-Ulloa et al., 2020). This suggested that those two metabolic types were likely to be the predominant forms utilized by microorganisms in deep-sea habitats (Oliveira et al., 2010). Similar predominant function categories related to the carbon cycle were observed in all sampling sites, which might be explained by the same dominant phylum appeared. Because composition and capabilities of heterotrophic microbial communities play a critical role in the detrital carbon cycle in sediments (Teske et al., 2011). Chloroflexi has potential to degrade a wide range of organic carbon, even harbored pathways for the complete hydrolytic or oxidative degradation of various recalcitrant organic matters (Liu et al., 2022). α-Proteobacteria could utilize a variety of organic compounds, mainly including amino acids, nucleic acids, fatty acids and other low molecular weight compounds, as well as organic and aromatic hydrocarbons produced by algae (Buchan et al., 2014; Mishamandani et al., 2016). γ-Proteobacteria as an opportunistic bacterial group was known to secrete extracellular enzymes to degrade polymers, nucleic acids and lipids, as well as refractory inert organic polycyclic aromatic hydrocarbons (Ivars-Martinez et al., 2008; Neumann et al., 2015). Crenarchaeota had genes for using carbohydrates as an organic carbon source, and genes for transporting amino acids from the environment (Parada et al., 2016). Those microbial groups were very likely worked together, contributing to the degradation of marine organic matter in the sediments of the Mariana Trench.

### Microbial metabolic activity

Incubation of EcoPlate™ provided a visual representation of the response rate of microbial community to different substrates and the final level achieved (Tian and Wang, 2011). In this study, the microbial community structure and microbial metabolic activity, reflected by richness index and the Shannon index, at deep stations was significantly different from those at shallow stations (except for Stn. TY041). This difference of microbial metabolic activity might be caused by significant differences in community composition between the deep and shallow stations, since the importance of sampling depth on the community composition and carbon source utilization had been observed in the sediments of the Arctic, Yap Trench and SCS (Hoffmann et al., 2017; Zhang C. et al., 2021; Zhang Y. et al., 2021). In addition, this difference might reflect the distinction of organic detritus sediments at different depths, including organic matter content, median grain size and silt-clay content (Zhang Y. et al., 2021). The shallowest station of Stn. TY035 had the lowest utilization of various carbon sources, especially carbohydrates. This might be due to the degradation of readily available dissolved organic carbon (DOC) in the upper water, while the remaining carbon source settled down to the bottom along the channel slope, confirmed by the low TOC content at shallow stations.

It was clear that the preferentially utilized organic matter during incubation was those could be easily degraded, because these compounds were considered to be the largest bioavailable source of carbon in sediments (Oliveira et al., 2010). In this study, polymers and carbohydrates were used preferentially, followed by carboxylic acids and amino acids. This might be due to the high concentration of organic matter belonging to polymers and carbohydrates in the sediments and the high proportion of microorganisms such as Chloroflexi and Proteobacteria utilizing these as carbon sources (Oliveira et al., 2010; Hoffmann et al., 2017; Zhao et al., 2020). As highly available carbon sources, amino acids were preferred by microorganisms because they could be transported into cells with high affinity (Grzymski and Dussaq, 2012).

The differences in microbial structure and function resulting from different depths create a differentiation of ecological niches, allowing various microorganisms to make full use of the limited resources in the deep sea. It should be noted that the washing efficiency of cells in sediments might cause differences in metabolic capacity between shallow and deep stations, although same operation procedures were applied. In addition, the types of carbon sources contained in microplate were limited and only provide a glimpse of the carbon source preference, and might not able to match the real *in situ* carbon source compositions in the natural sampling locations. In the future, isotopic tracing experiments together with meta-transcriptomics would help to elucidate the real process of carbon source utilization by microbes occurred in the trenches.

## Data availability statement

The datasets presented in this study can be found in online repositories. The names of the repository/repositories and accession number(s) can be found at: NCBI, PRJNA854746.

## Author contributions

HJ conceived and designed the experiment. FW performed the experiment and wrote the first draft. YZ and FW analyzed the data. HJ, YZ, and HL revised the manuscript. All authors contributed to the article and approved the submitted version.

## Funding

This work was supported by the Hainan Province Science and Technology Special Fund (ZDKJ2021036 and ZDKJ2019011), the Hainan Provincial Natural Science Foundation of China for High-level Talents (420RC677), and the National Natural Science Foundation of China (41776147).

## Conflict of interest

The authors declare that the research was conducted in the absence of any commercial or financial relationships that could be construed as a potential conflict of interest.

## Publisher’s note

All claims expressed in this article are solely those of the authors and do not necessarily represent those of their affiliated organizations, or those of the publisher, the editors and the reviewers. Any product that may be evaluated in this article, or claim that may be made by its manufacturer, is not guaranteed or endorsed by the publisher.

## Footnotes

^1^ http://huttenhower.sph.harvard.edu/galaxy/
