# Supplementary material for: Spatial variation and metabolic diversity of microbial communities in the surface sediments of the Mariana Trench
Source: Front Microbiol. 2022 Dec 5;13:1051999. doi: 10.3389/fmicb.2022.1051999 (PMC9760864; doi:10.3389/fmicb.2022.1051999)
Supplement: Supplementary file 1 [file Data_Sheet_1.docx]

**Supplementary materials:**

**Table S1.** The chemical parameters of sediments collected from the Mariana Trench.

|  | Station | Depth  (m) | TP  (mg/kg) | NO_3_-N (mg/kg) | NH_4_-N (mg/kg) | TN (%) | TOC  (%) | C/N | Moisture  (%) |
| --- | --- | --- | --- | --- | --- | --- | --- | --- | --- |
| shallow | TY035 | 6,957 | 445.90 | 0.57 | 0.70 | 0.03 | 0.22 | 7.68 | 1.90 |
|  | TY048 | 7,329 | 1,571.78 | 9.22 | 2.59 | 0.04 | 0.18 | 5.11 | 0.14 |
|  | TY044 | 7,344 | 1,293.53 | 9.22 | 3.13 | 0.04 | 0.18 | 5.11 | 0.14 |
|  | TY041 | 8,563 | 1,578.82 | 9.92 | 2.93 | 0.03 | 0.22 | 8.44 | 0.15 |
| deep | TY042 | 10,109 | 1,044.60 | 11.59 | 1.30 | 0.05 | 0.47 | 8.97 | 0.13 |
|  | TY038 | 10,893 | 926.58 | 14.03 | 0.98 | 0.05 | 0.40 | 7.94 | 2.47 |
|  | TY039 | 10,910 | 1,028.97 | 30.24 | 1.95 | 0.05 | 0.37 | 7.85 | 3.24 |
|  | TY046 | 10,918 | 1,334.96 | 7.45 | 0.97 | 0.03 | 0.25 | 7.42 | 2.67 |


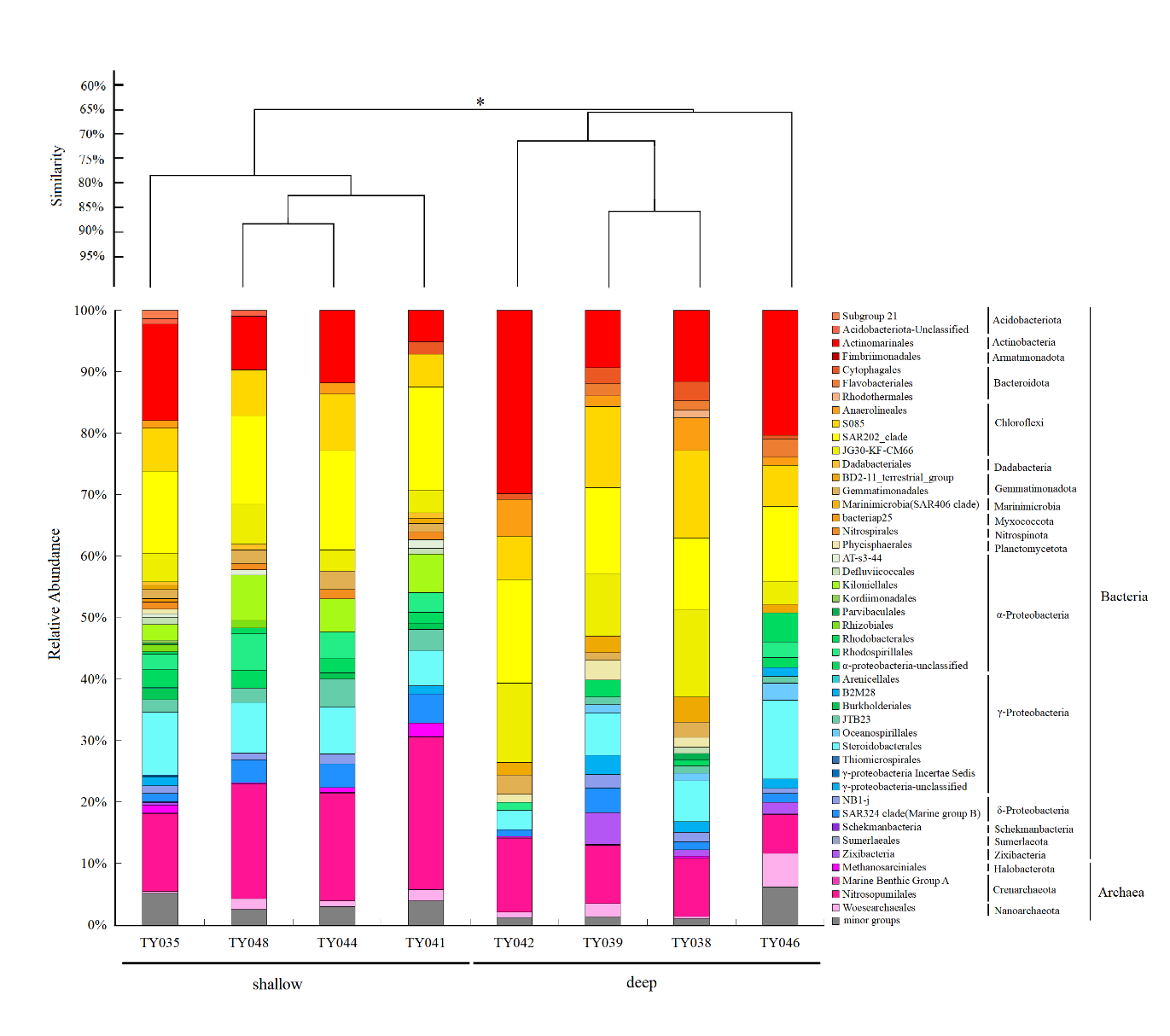


**Figure S1.** Microbial community structure of different sediment samples with clustering at the order level. * *p* < 0.05, those with relative abundance less than 1% were classified as minor groups.
